# A shape control and object manipulation technique based on function approximation for robotic surfaces

**DOI:** 10.3389/frobt.2025.1633131

**Published:** 2025-11-05

**Authors:** Yuchen Zhao, Yuxin Chen

**Affiliations:** 1 School of Automation, Southeast University, Nanjing, Jiangsu, China; 2 Ministry of Education Key Laboratory of Measurement and Control of Complex Systems of Engineering, Southeast University, Nanjing, Jiangsu, China

**Keywords:** robotic surfaces, object manipulation, cellular robots, distributed robot systems, pin array, refresh time scaling

## Abstract

Robotic surfaces consisting of many actuators can change shape to perform tasks, such as object transportation and sorting. Increasing the number of actuators can enhance the robot’s capacity, but controlling a large number of actuators is a challenging problem that includes issues such as the increased system-wide refresh time. We propose a novel control method that has constant refresh times, no matter how many actuators are in the robot. Having a distributed nature, the method first approximates target shapes, then broadcasts the approximation coefficients to the actuators and relies on itself to compute the inputs. To confirm the system size-independent scaling, we build a robot surface and measure the refresh time as a function of the number of actuators. We also perform experiments to approximate target shapes, and a good agreement between the experiments and theoretical predictions is achieved. Our method is more efficient because it requires fewer control messages to coordinate robot surfaces with the same accuracy. We also present a modeling strategy for the complex robot–object interaction force based on our control method and derive a feedback controller for object transportation tasks. This feedback controller is further tested by object transportation experiments, and the results demonstrate the validity of the model and the controller.

## 1 Introduction

Robotic surfaces (Liu et al., 2021; Walker, 2017) typically consist of many actuation modules arranged in an array and can serve as intelligent conveyors (Uriarte et al., 2019; Chen et al., 2024), adaptive structures (Wang et al., 2019; Salerno et al., 2020), molding tools (Tian et al., 2022), treadmills (Smoot et al., 2019), shape displays, or haptic interfaces (Leithinger et al., 2014; Nakagaki et al., 2019). The capability of a robotic surface is related to the number of actuators it has as the robot can perform multiple tasks in parallel with more actuators. Developments in soft robotics also bring new solutions to meet the demand of actuators (Liu et al., 2021; Johnson et al., 2023; Robertson et al., 2019). However, coordinating many actuators is challenging. Generating control commands for them requires a large amount of resources, such as physical space, equipment, and communication bandwidth (Winck and Book, 2013). A noticeable quantity is the time delay between the first and the last actuator when updating the system to a new shape, which we note as the refresh time τ . A small τ is preferable in real-time tasks. Standard communication methods send control messages to each actuator in a sequential fashion (Follmer et al., 2013; Xue et al., 2024; Siu et al., 2018; Stanley et al., 2016; Leithinger and Ishii, 2010), so τ has an O(N) scaling where N is the number of actuators. To control more actuators, strategies such as using multiple communication channels, sharing one motion controller in a small group of motors, or performing multithreading in the central computer have been employed (Johnson et al., 2023; Follmer et al., 2013; Xue et al., 2024; Siu et al., 2018). By optimizing system architectures, these methods are helpful in reducing refresh time, but the fundamental scaling relation of the refresh time remains unchanged, and the scalability can be improved.

A non-sequential and more scalable approach is to drive each row and column of the actuator array, similar to the matrix drive technique used in LED displays (Chen et al., 2011). This method simultaneously controls all actuators on the same row or column. Early works from Zhu, Winck, and Book et al. show that the scaling of τ can be reduced to $O\left(\sqrt{N}\right)$ . They developed a control loop structure based on singular value decomposition that can drive a hydraulic cylinder robotic array to any shape via $2\sqrt{N}$ row and column controllers (Zhu and Book, 2004; 2006; Winck and Book, 2012; Winck et al., 2012; Winck and Book, 2017). In this method, a “control coupler” valve is needed for each cylinder to couple the row and column control signals (Ferguson et al., 2020). In soft robotics, a compact fluidic logic module is designed to regulate the input row and column pressures for a pneumatic soft linear actuator array (Jadhav et al., 2023). In addition, a robotic surface is fabricated with ionic polymer stripes and controlled using peripheral voltages (Wang et al., 2023). However, these methods also lack scalability because the τ is still dependent on system size. The τ scaling issue can become more severe when the area density of actuators increases.

**FIGURE 1:**
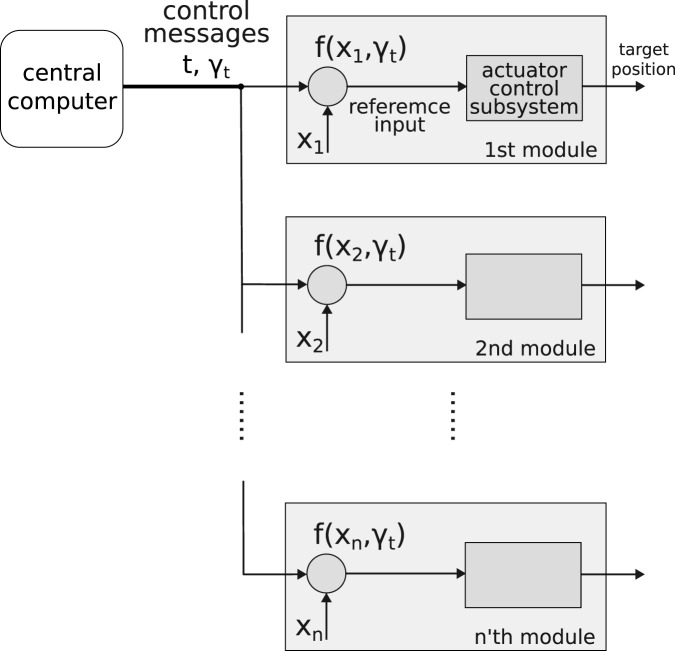
An illustration of our control method. At time t , the control message $\gamma_{t}$ is broadcast to all modules. In the nth module, the reference input is calculated via a function f and its arguments $\gamma_{t}$ and $x_{n}$ . This input is sent to an actuator subsystem that generates a targeted position.

We propose a novel control method for robotic surfaces that has a system size-independent refresh time. In general, a continuous surface profile is first discretized, and the relevant actuation commands are passed to each actuator. Our method focuses on the second part of shape control, so we simply discretize the surface profile on a square lattice representing the pin array. When approximating the discretized surface profile, it is worth noting that (1) neighboring actuators usually have similar inputs, so there is no need to send the inputs to every one of them; (2) the discretized surface profile may be simply parameterized, such as Gaussian function-like patterns used in object manipulation tasks (Johnson et al., 2023), in which only two center coordinates are important. Therefore, in our method, a central computer broadcasts features of the target shape to individual actuation modules and allows them to calculate their inputs. Our method is illustrated in Figure 1. This approach results in a size-independent scaling O(1) of the refresh time, and the residual error of shape approximation becomes dependent on the target shape and approximation algorithm. We experimentally validate the refresh time scaling on a 4×4 pin array setup and compare it to the sequential control method. In order to achieve any shape, we use function forms with universal approximation properties and employ the discrete cosine transform (DCT) and the matching pursuit (MP) algorithms (Mallat and Zhang, 1993) to compute shape features. We further characterize shape change capability by displaying six distinct shapes and measuring their height profiles. In addition to the improvement in the refresh time scaling, our control method allows an interpretable modeling procedure for the complex robot–object interaction force F . Based on the force model, we design a feedback controller for object transportation tasks and perform experiments to confirm its validity.

The rest of the article is organized as follows. In Section 2, we present the control method and algorithms to compute the shape features, derive equations for the object manipulation problem, and describe the robot setup. In Section 3, we present our experimental results on the time-delay scaling, quantification of shape-changing capacity, and object manipulation tasks. Section 4 contains a discussion and concluding remarks.

## 2 Materials and methods

### 2.1 Control message calculation

Our control method is illustrated in Figure 1. One highlight is that the control messages $\gamma_{t}$ are broadcast to all actuation modules at each time step t to eliminate communication time delays between the modules. In our case, an actuation module refers to an independent system that includes an actuator, a sensor, and microcontrollers (see Section 2.3). The actuator control subsystem is responsible for regulating the state of each actuator to a given reference so that all actuators can represent a given surface shape. $\gamma_{t}$ is a set of shape feature parameters used by each module to compute the reference input for the actuator control subsystem. To be able to generate any target shape using broadcast control message $\gamma_{t}$ , we utilize function forms with universal approximation ability. To be more specific, once the modules receive the same $\gamma_{t}$ , they can use a single function form $f\left(x_{n},\gamma_{t}\right)$ with universal approximation properties and a local parameter $x_{n}$ to compute their inputs, making it possible for the robot to approximate any target shape. In practice, the function forms are stored on each module microcontroller board for computing the input, and $x_{n}$ is an identification value stored on the nth module.

The universal approximation ability guarantees that any target shape can be exactly represented by a set of $\gamma_{t}$ . Depending on the function forms, different algorithms exist for computing $\gamma_{t}$ . We use two function forms to test the refresh time scaling and shape-changing ability. We first choose the cosine function because we can use off-the-shelf discrete cosine transform (DCT) algorithms to compute the shape features as $\gamma_{t}$ . The input $f_{n}$ to the actuator control subsystem in the nth module is represented as:

$$f_{n}=a_{0}+2\overset{N-1}{\underset{t=1}{\sum }}a_{t}cos\left(k_{t}\left(2x_{n}+1\right)\right),\;k_{t}=\frac{\pi t}{2N}$$ (1)

where $a_{t}$ and $k_{t}$ are the amplitude and the wave vector at time t , and $\gamma_{t}=\left(a_{t},k_{t}\right)$ . The shape features $a_{t}$ and $k_{t}$ are computed using the DCT algorithm from the Scipy package fftpack. To construct an accurate target shape, several $\gamma_{t}$ are broadcast to all modules. Upon receiving the messages, each module computes the input $f_{n}$ according to Equation 1 with its own $x_{n}$ . There is no system size-dependent refresh time because all modules can simultaneously receive $\gamma_{t}$ and compute $f_{n}$ in parallel.

Considering that the cosine function may not be efficient in representing localized patterns, we use time–frequency functions to capture both extended and localized patterns:

$$f_{n}=\overset{\infty }{\underset{t=0}{\sum }}a_{t}g_{\sigma_{t}}\left(x_{n}-p_{t}\right)\cos\left(\frac{2\pi k_{t}}{N}x_{n}+\phi_{t}\right)$$ (2)

where $g_{\sigma }\left(x\right)=\sum_{j=-\infty }^{+\infty }\exp\left(-\pi {\left(\frac{x-jN}{\sigma }\right)}^{2}\right)$ is a Gaussian function made periodically on the domain [0,N] . The coefficients in $\gamma_{t}=\left(a_{t},\sigma_{t},p_{t},k_{t},\phi_{t}\right)$ are the amplitude, scale, position, frequency, and phase of the time–frequency function, respectively. If $\sigma_{t}$ is large, and $g_{\sigma }\left(x\right)\approx 1$ over all x in the domain, then Equation 2 reduces to the cosine functions in Equation 1. To compute $\gamma_{t}$ for any target shape, we use the matching pursuit algorithm (MP) for a dictionary of time-frequency atoms. The details of the algorithm can be found in Mallat and Zhang (1993). We implement the MP algorithm in Python. It takes a given pattern ${\left\{f_{n}\right\}}_{n=1}^{N}$ and computes ${\left\{\gamma_{t}\right\}}_{t=0}^{M}$ sequentially to some prescribed M . The reconstructed pattern using finite terms converges to the original one exponentially fast, as proven in Mallat and Zhang (1993). In practice, we find that only a few terms are needed to approximate a pattern with good accuracy (see Section 3.2).

For object manipulation tasks, we use the Gaussian radial basis function (GRBF):

$$f_{n}=\overset{\infty }{\underset{t=0}{\sum }}a_{t}\exp\left(-\frac{{\left(x_{n}-d_{t}^{\left(x\right)}\right)}^{2}+{\left(y_{n}-d_{t}^{\left(y\right)}\right)}^{2}}{\sigma_{t}^{2}}\right)$$ (3)

where the coefficients in $\gamma_{t}=\left(a_{t},\sigma_{t},d_{t}^{\left(x\right)},d_{t}^{\left(y\right)}\right)$ are the amplitude, width, and center coordinates of each Gaussian function. $\left(x_{n},y_{n}\right)$ are two identification values stored on the nth module, representing the 2D physical coordinates of the module in the array. Although the GRBF is capable of universal approximation (Park and Sandberg, 1991), algorithms computing $\gamma_{t}$ may require time-consuming optimization. For this reason, we do not use this function form in shape-changing experiments. Instead, we use the GRBF in object manipulation tasks, and one term in Equation 3 is sufficient.

### 2.2 Force model and controller design

Generally speaking, an object is governed by the equation of motion of its center of mass $F=m\ddot{X}\left(t\right)$ , where m is the object mass, and F is the total force acting on the object. F includes the object’s gravity, and, importantly, the nonlinear interactions between the object and the robotic surface. Because the surface usually involves many degrees of freedom, determining a sequence of shapes to manipulate an object is a difficult task. Previous works achieve object manipulations with empirical relations (Johnson et al., 2023; Chen et al., 2021) or black-box machine learning models (Wang et al., 2023; Xue et al., 2024), both of which require data for calibration. Our proposed control method utilizes shape features to generate surfaces, therefore effectively reducing the computational burden of searching for the right shapes. Furthermore, the shape we used allows us to build a reasonable force model to guide feedback controller design for object manipulation tasks; hence, no training data are needed to design the controller.

**FIGURE 2:**
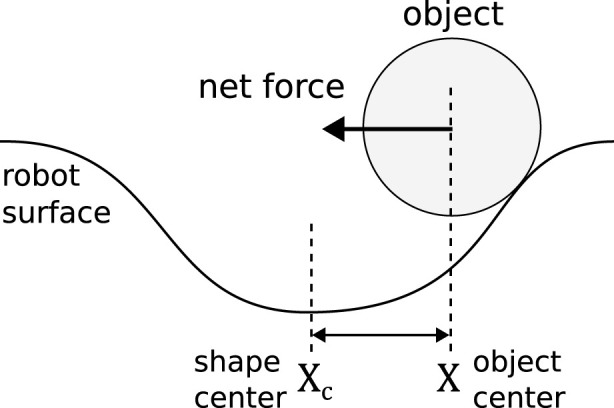
An illustration of the idealized object–robot surface interaction.

In this work, we manipulate an object with a shape defined by one concave (a<0) GRBF in Equation 3 and a time-varying center coordinate $X_{c}\left(t\right)≔\left(d^{\left(x\right)},d^{\left(y\right)}\right)$ . The robot shape control problem is drastically reduced to determining two scalars contained in $X_{c}\left(t\right)$ . We first derive the force model. We assume that the surface is smooth, the object is always in contact with the surface, and the net force is purely determined by the static geometric configurations of the shape and the object. The case is illustrated in Figure 2. Depending on the local shape gradient, the object experiences a small net force either near or far away from the shape center $X_{c}\left(t\right)$ , and a large net force when $\left\|X\left(t\right)-X_{c}\left(t\right)\right\|$ is comparable to the width of the Gaussian σ . Hence, the magnitude of F can be expressed as

$$\|F\|=g\left(\frac{\left\|X\left(t\right)-X_{c}\left(t\right)\right\|}{\sigma }\right),$$ (4)

where g(s) is a single-peak function that takes the maximum value at s=1 and remains zero when s≈0 and s≫1 . Furthermore, because the GRBF has radial symmetry, the direction of F points to the shape center $X_{c}\left(t\right)$ :

$$\hat{F}=\frac{X_{c}\left(t\right)-X\left(t\right)}{\left\|X_{c}\left(t\right)-X\left(t\right)\right\|}.$$ (5)

Equations 4, 5 together provide a force model when using a single GRBF to generate the shape.

$$F=\frac{X_{c}\left(t\right)-X\left(t\right)}{\left\|X_{c}\left(t\right)-X\left(t\right)\right\|}g\left(\frac{\left\|X\left(t\right)-X_{c}\left(t\right)\right\|}{\sigma }\right).$$ (6)

The force model is obtained via order-of-magnitude analysis and a symmetry consideration; hence, many realistic details are not captured, such as the discreteness of the robot shape due to finite actuator size, object rotation, friction, and visco-elastic force at contact. We also focus on the planar movement of the object and ignore its vertical motion.

The feedback controller needs to compute the shape center $X_{c}\left(t\right)$ based on the object center X(t) and the target location $X_{\text{ref}}$ . Based on the force model in Equation 6, two heuristics can be used to guide the design of the controller. First, we want to maintain the net force direction $\hat{F}$ toward the target position $X_{\text{ref}}$ , so the object will not go elsewhere. Moreover, we should keep $X_{c}\left(t\right)$ next to X(t) , so the driving force F does not vanish. Mathematically, we first demand that F point toward $X_{\text{ref}}$ . This constraint is expressed as

$$X_{c}\left(t\right)-X\left(t\right)=\lambda \left(X_{\text{ref}}-X\left(t\right)\right),$$ (7)

where λ determines the magnitude of $X_{c}\left(t\right)$ . Based on the assumption that g(s) takes the maximum value at s=1 , we can demand

$$\|X_{c}\left(t\right)-X\left(t\right)\|=\sigma$$ (8)

so that ‖F‖ attains its greatest value. Equations 7, 8 together determine $X_{c}\left(t\right)$ :

$$X_{c}\left(t\right)=\left(\lambda -1\right)\left(X_{\text{ref}}-X\left(t\right)\right)+X_{\text{ref}}$$ (9)

$$\lambda =\frac{\sigma }{\left\|X_{\text{ref}}-X\left(t\right)\right\|}.$$ (10)

**FIGURE 3:**
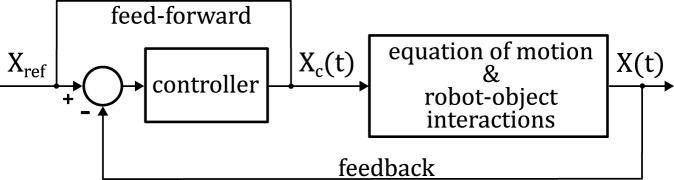
A control block diagram for the object manipulation task.

The first and second terms (Equation 9) are the proportional feedback and feed-forward control, respectively. The control loop is shown in Figure 3.

To ensure control process stability when X(t) approaches $X_{\text{ref}}$ and λ becomes large, we restrict λ to [0,1], so that $X_{c}\left(t\right)=X_{\text{ref}}$ whenever $\|X_{\text{ref}}-X\left(t\right)\|\leq \sigma$ . This nonlinear constraint replaces (10) and can be expressed as

$$\lambda =\min\left(\frac{\sigma }{\left\|X_{\text{ref}}-X\left(t\right)\right\|},\;1\right),$$ (11)

The controller only has one parameter σ to tune. In fact, σ should be comparable to the characteristic size of the object, leaving no additional parameter to adjust. Even though the force model is under many assumptions, the controller derived from it can accomplish object transportation tasks, as demonstrated in the experiments (Section 3.3), showing that this procedure is effective. However, as there is no explicit incorporation of disturbance rejection mechanisms, its performance may be compromised if the object–surface interactions largely deviate from ideal assumptions, as shown in Section 3.3. In practice, the control loop functions as follows: first, the position of the object X(t) is captured by a camera, and this information is sent to the central computer to compute the shape center $X_{c}\left(t\right)$ according to Equations 9, 11. The central computer then sends $X_{c}\left(t\right)$ along with other shape features required by the GRBF (Equation 3) to the robot, and the robot displays the shape to manipulate the object. The GRBF is not used in the feedback controller. Details of the hardware implementation are described in Section 2.3.

### 2.3 Experimental setup

**FIGURE 4:**
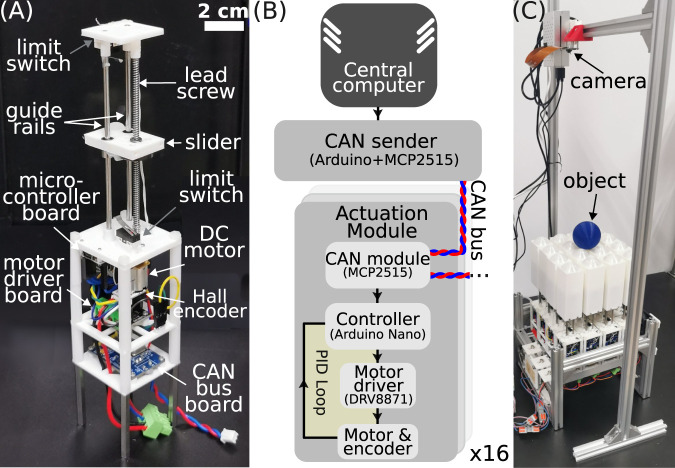
**(A)** A picture of a single linear actuation module. The rectangular cover is removed to expose mechanical components. **(B)** Block diagram of the electronic system. The arrows indicate information flows. **(C)** The robot and vision servo system for object manipulation.

The robot has a modular design and consists of 16 identically built linear actuation modules arranged in an 85-mm-long square area. As shown in Figure 4A, the module is approximately 47 mm wide and 200 mm long. A lead screw of 100 mm in length and 2.5 mm in pitch converts the rotary motion of a DC motor to linear motion. The screw is attached to a slider with two additional guide rails parallel to the lead screw to reduce friction. Two limit switches are installed at the two ends to prevent overshoot that may damage the motor, and the overall arrangement of mechanical parts results in a linear stroke of 70 mm. A complete module also has a rectangular cover attached to the slider (see Figure 4C). The DC motor (Tianqu Motor, N20VA, 1:10) has a rated maximum speed of 50 revolutions per second, leading to a nominal speed of 125 mm/s of the linear motion. The motor’s tail has a Hall rotary encoder to measure the angular position of the shaft, defined as $e_{n}$ for the nth module. The vertical position $h_{n}$ of the linear actuator is proportional to $e_{n}$ . All mechanical components and electronics are mounted on 3D printed frames, and the modules are mounted on a portable aluminum frame. When varying the system size N , we simply connect or disconnect modules from the robot.

The electronics block diagram is shown in Figure 4B. The controller of the actuator is an Arduino Nano board, which is programmed as a closed-loop control system for shaft position $e_{n}$ and can receive control messages from a central computer. A PID controller running every 16 ms (62.5 Hz) is in use. Two additional modules are connected to the controller board. A DRV8871 board is used to drive the motor with the standard pulse width modulation (PWM) technique. An MCP2515 CAN bus board is used for receiving control messages. All actuation modules are on one CAN bus, and the protocol is CAN 2.0B. While identical in hardware, there is a unique identification variable $x_{n}$ ranging from 0 to 15 in the nth module’s software. This variable is involved in reference input calculation in Equations 1–3, or acts as the CAN message identifier in the sequential control method. A central computer (Raspberry Pi 4B) is used to generate target shapes $\left\{f_{n}\right\}$ and the control messages $\left\{\gamma_{t}\right\}$ to drive the modules. In our method, we broadcast a single $\gamma_{t}$ in one standard CAN data frame (which holds 8 bytes of data), meaning the coefficients are represented with limited resolution. The data frame also contains information on which function form to use, so we can switch between different approximation methods. An additional Arduino Nano and an MCP2515 module serve as the CAN bus sender that interfaces with the central computer and the modules.

For the object manipulation tasks, a simple vision servo system is built to track the object and compute the control output $X_{c}\left(t\right)$ to drive the robot. The setup is shown in Figure 4C. A web camera (Raspberry Pi Camera V1) is mounted above the robot and faces downward to capture an image sequence at a rate about 22 Hz. Each image contains a colored object and the actuation modules as the background. The object coordinate X(t) is the center of mass of the largest connected component in a binarized image, and we use a color threshold to identify the object. All images are processed with OpenCV in Python. A calibrated 2D affine transform is used to relate the object coordinates in images to those in the lab. Given X(t) and the target position $X_{\text{ref}}$ , the new shape center position $X_{c}\left(t\right)$ is computed according to Equations 9, 11. We reduce noise in $X_{c}\left(t\right)$ with a moving average filter of size 15 to reduce actuator jittering. The smoothed $X_{c}\left(t\right)$ is sent to the robot for actuation, and the control loop update frequency is the same as that of the image.

## 3 Results

### 3.1 Experiments on refresh time

**FIGURE 5:**
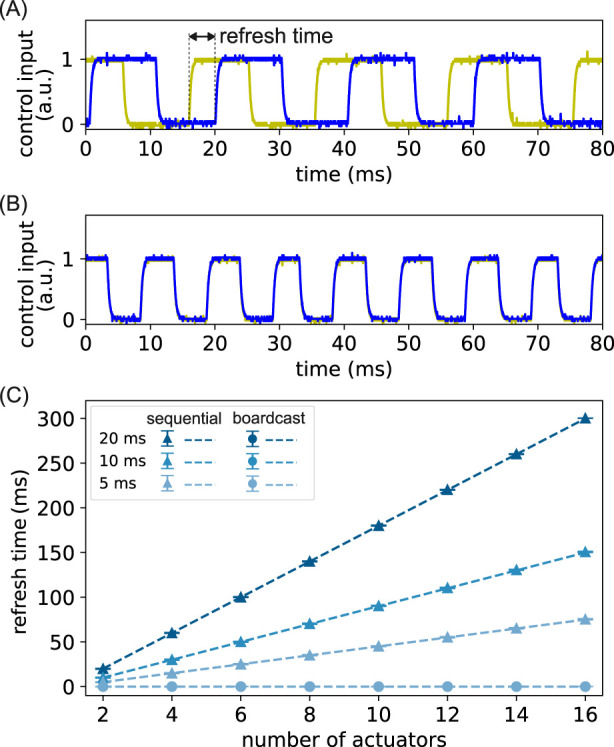
Experimental refresh time scaling without actuator dynamics. **(A)** The reference inputs of two actuators when using a sequential control method. The yellow (blue) line is the first (last) actuator in a two-actuator system; **(B)** the reference inputs of the same actuators when using our control method; **(C)** the averaged refresh time is plotted as a function of the number of actuators for the two control methods and at different communication rates (expressed in $T_{\text{msg}}$ ). Each point is an average of at least 20 refresh time values observed in **(A)** or **(B)**. The darker color represents larger $T_{\text{msg}}$ . The triangles (circles) are experimentally measured refresh times with the sequential (our) control method, and the dashed lines are theoretical predictions.

We first perform an experiment to measure the refresh time where there is no actuator dynamics and only communication delay between the central computer and the actuator modules. The robot is refreshed between two uniform patterns ${\left\{f_{n}=0\right\}}_{n=1}^{N}$ and ${\left\{f_{n}=1\right\}}_{n=1}^{N}$ at a constant rate using either the standard sequential control or our method described in (1). Note that because all actuators have equal $f_{n}$ values, only one CAN message is needed. To measure $f_{n}$ on each actuator, we correlate the variable with a PWM output on the controller board and convert the PWM output using a digital-to-analog converter based on an LCR low-pass circuit. The signals of the first and the Nth actuation module are simultaneously measured on an oscilloscope (Rigol DS1202EZ), and the time delay is extracted. We vary N from 2 to 16. Typical signals are shown in Figure 5A and (B) for a system with N=2 actuators. We extract the refresh time using normalized cross-correlation and average over at least 20 values.

The average refresh time is plotted as a function of N for both methods in Figure 5C. As expected, for the sequential control method, the refresh time shows a linear increasing trend, and the slope equals the time period $T_{\text{msg}}$ to send one control message. We vary $T_{\text{msg}}$ from 5 ms to 20 ms, and the predicted trends agree well with experimental results. In contrast, for our method, the refresh time remains at zero for all N and all $T_{\text{msg}}$ , showing that there is no time delay among the modules due to system size or communication delay. Therefore, we confirm that our method indeed achieves a system size-independent refresh time when no actuator dynamics is present.

**FIGURE 6:**
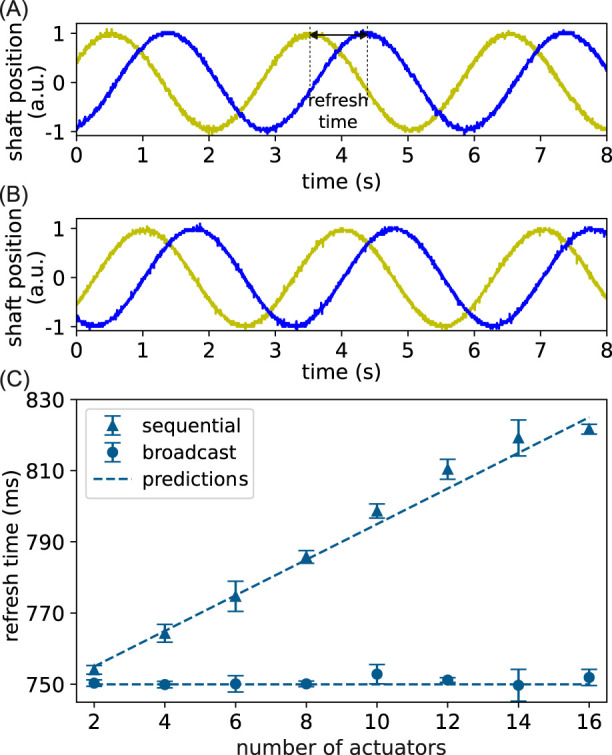
Experimental refresh time scaling with actuator dynamics. **(A)** The motor shaft angular position when using the sequential control method. The yellow (blue) trace is the first (last) actuator in a 16-actuator system, with its refresh time indicated in the dashed line; **(B)** the shaft position of the same actuators when using our control method. **(C)** Refresh time is plotted as a function of the number of actuators for the two control methods. The triangles and circles are experimentally measured refresh times using the sequential and our control methods, respectively. Each point is an average of at least six refresh times, and the error bar is one standard deviation. The dashed lines are theoretical predictions.

In the second experiment, we take the actuator dynamics into account by measuring the refresh time between the shaft positions $e_{n}$ of the first and the last actuator. In addition to the above, the robot is refreshed with a traveling wave pattern, so the refresh time is also determined by the wave speed v . The traveling wave is a quarter of a moving sinusoid over N actuators, given by

$$f_{n}=\sin\left(k_{N}x_{n}-vt\right)$$ (12)

where $k_{N}=\frac{\pi }{2\left(N-1\right)}$ is the system-size-dependent wave vector chosen such that the last actuator $x_{N}=N-1$ always has a quarter phase, $k_{N}x_{N}=\frac{\pi }{2}$ . t is the current time. The control message is sent every $T_{\text{msg}}=5$ ms from the central computer. For the sequential control method, $f_{n}$ is computed and transmitted to each actuator. For our method, we express $f_{n}$ using a simplified version of Equation 2 because Equation 12 can be rewritten as $f_{n}=a\cos\left(k_{N}x_{n}-\pi /2\right)+b\cos\left(k_{N}x_{n}\right)$ , where a=cos(vt) and b=−sin(vt) . The coefficients $k_{N}$ , a , and b are broadcast to all modules in one CAN data frame. The observed traveling waves for both methods are shown in Figures 6A,B, and the average refresh time τ as a function of the number of actuators N is shown in Figure 6C. For our method, τ stays constant for all N , hence validating the system size-independent refresh time when both signal transmission and actuator dynamics are present. According to Equation 12, the refresh time between the first and the last actuator should be the time to catch up their phase difference, that is, $\tau =k_{N}\left(x_{N}-x_{1}\right)/v=\pi /\left(2v\right)$ . In all experiments, we set v=2π/T and T=3000 ms, so τ=T/4=750 ms. Our experimental results in Figure 6C agree with this value, with a relative error less than 1% for all N , and the discrepancy is likely due to random errors. For the sequential control method, τ is larger than the theoretical 750 ms and linearly increases as N increases. When one module is added to the system, it takes an additional amount of time to transmit control messages to that module, so the refresh time for the sequential control method is $\tau_{\text{seq}}=T/4+T_{\text{msg}}\left(N-1\right)$ . This prediction is plotted as a dashed line in Figure 6C, which also agrees with the experimental results.

### 3.2 Characterization of shape change

**FIGURE 7:**
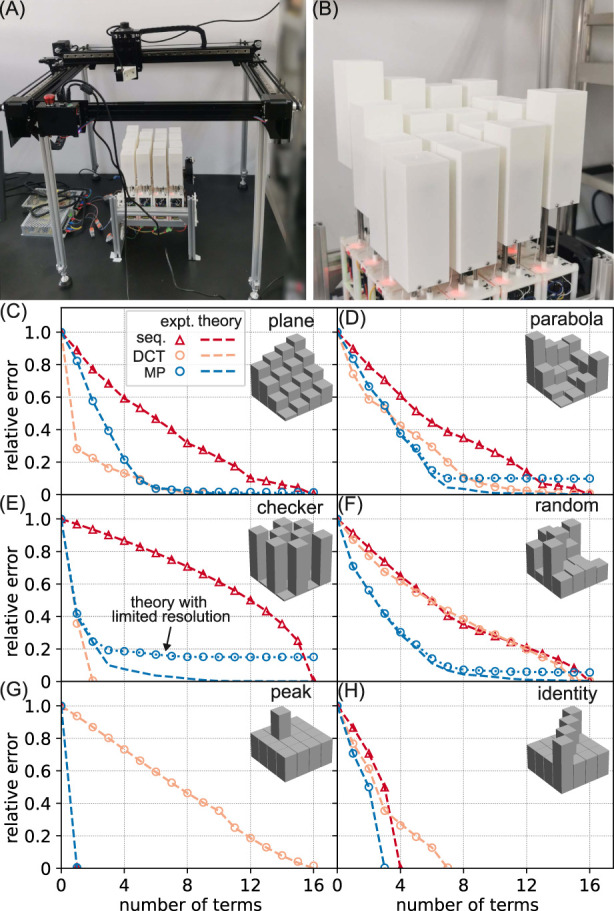
Characterization of shape-changing ability. **(A)** The experimental setup for shape measurement; **(B)** the parabola shape displayed by the robot; **(C–H)** the relative error of the shape is plotted as a function of the number of terms used to approximate the shape. The triangles (circles) are from the sequential (our) control method. The orange and blue colors of the circles correspond to the DCT and MP algorithms, respectively. Each data point is an average of three independent runs. The error bar is smaller than the marker size, so it is not shown. The dashed lines are theoretical predictions calculated with 64-bit floating point numbers, and the dotted blue lines are calculated with 16- and 8-bit resolution-limited numbers. The inset displays the target shape.

To quantify the approximation accuracy of our control method, we drive the robot to six distinct shapes and quantify the errors between the target and the measured shapes. The shape measurement apparatus is shown in Figure 7A. A laser distance meter (Shanghai Kedi, KG01) is used to measure the height change of each actuator, and the scan process is automated via a homemade Cartesian robot. The system has an accuracy of 0.2 mm. The six shapes are listed in Table 1. All shapes are represented as a 4×4 matrix, where the elements are actuator vertical positions $h_{n}$ . The actuation module’s planar coordinates $\left(x_{n},y_{n}\right)$ are taken from {0,1,2,3} , and we stretch the vertical scale so that each shape can fill the entire 70-mm stroke of the actuator. The parabola shape displayed by the robot is shown in Figure 7B, and all shapes are illustrated as the insets in Figures 7C–H. For each run of the experiment, we start with the robot leveled at half stroke length. Then, the robot is actuated through a series of intermediate shapes. Following each shape actuation, we perform a height scan to track the shape change and compute the relative error between the intermediate shape and the target shape using root mean squared error. The intermediate shape is achieved incrementally with additional information from one control message $\gamma_{t}$ that contains all coefficients in one approximation term in Equations 1, 2. For the sequential control method, the term is the reference vertical position $h_{n}$ of a single actuator. For our method, we test both function approximation formulas in Equations 1, 2. To compute the coefficients, we reshape the 4×4 shape matrix into a vector and apply the DCT or MP algorithm. For the order of shape actuation, we prioritize terms with a higher amplitude or $h_{n}$ values.

**TABLE 1:** Names and expressions of the six shapes.

Name	Expression	Name	Expression
Identity	a 4×4 identity matrix	Plane	z=x+2y
Parabola	$z=2x^{2}+3y^{2}-3xy$	Checkers	Checkerboard pattern
Peak	raise a single module at x=1,y=2	Random	Random uniform distribution

The relative error as a function of the number of terms (or equivalently, the number of control messages) is shown in Figures 7C–H. Our control method with the MP algorithm can outperform the sequential control method in the sense that it requires fewer terms to approximate the target shape for the same error. For example, it takes 11 terms for the sequential method to approximate the parabola shape to a 20% relative error, while it takes six and seven terms for the MP and DCT algorithms, respectively (Figure 7D). We also find that within our methods, MP can approximate both extended and localized patterns with fewer terms, while DCT approximates extended patterns better than localized patterns. The DCT algorithm outperforms the sequential method in three target shapes (plane, parabola, and checker) but can be worse for localized patterns. For example, for the peak shape, it takes only one term for MP to reach a perfect match, while DCT needs all 16 terms. The relative errors obtained from experiments are well captured by theoretical predictions based on Equations 1, 2, except in the parabola, checker, and random cases, where MP leads to residual errors that are not captured by Equation 2. These residual errors are due to the limited coefficient resolution used in computing the approximation terms. To include the five coefficients from Equation 2 into one CAN data frame, $s_{t}$ and $a_{t}$ are allocated with 2 bytes each, and $p_{t}$ , $k_{t}$ , and $\phi_{t}$ are 1 byte each. By recalculating intermediate shapes using these less precise numbers, we can match the experimental results, as shown by the dotted lines in Figures 7D–F.

### 3.3 Closed-loop object manipulation

We demonstrate the object manipulation capability of the robot based on our closed-loop control method derived in Section 2.2. Motion videos are provided in the Supplementary Material. The objects are 3D printed light-weight spheres of diameter $D_{\text{sphere}}=6,\;8$ cm and cubes of edge length $D_{\text{cube}}=6,\;8$ cm. A spherical cap is attached to the top of each rectangular cover to prevent the object from stabilizing itself on the cover. A single GRBF generates robot shape to drive the object, and its amplitude a=−D and width σ=D , where D is the object’s characteristic size and equals $D_{\text{sphere}}$ or $D_{\text{cube}}$ , so that the robot shape is comparable to the object size. A negative a implies a concave valley for holding the object. Although the object can remain stable inside the valley when the shape is frozen, our controller always changes the shape such that the object never establishes force equilibrium until the target position is reached. Note that our controller does not assume the shape of the object, and the only tunable parameter in the controller is σ , which we set to D .

**FIGURE 8:**
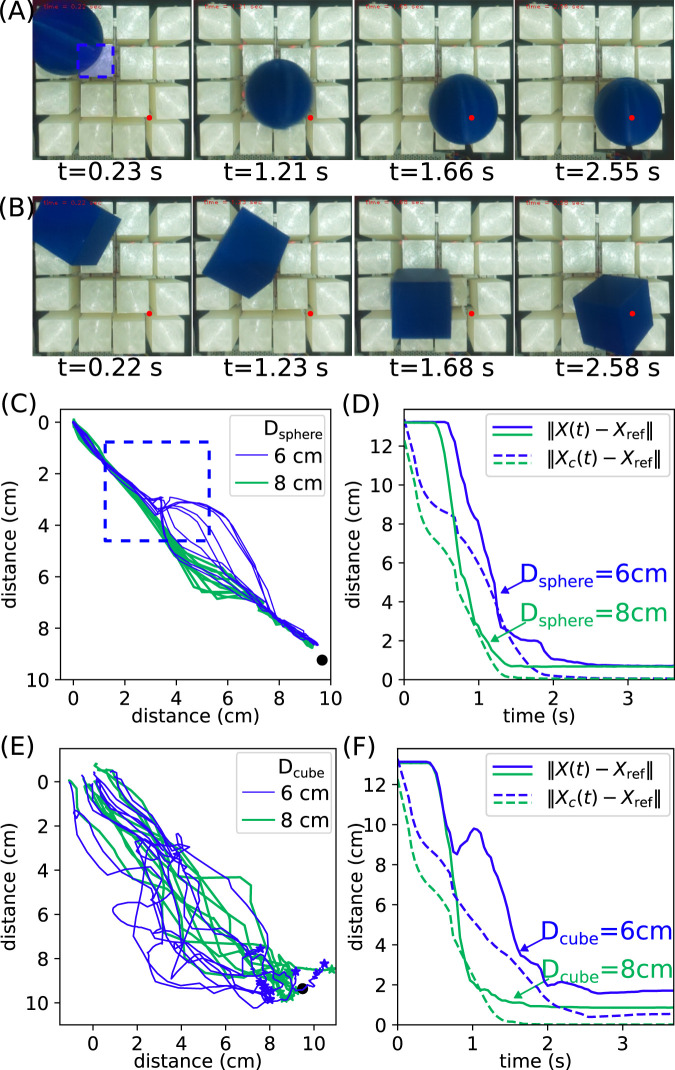
Object transportation experiments. **(A)** Snapshots of transportation processes of a sphere with $D_{\text{sphere}}=8$ cm. The target position is marked by a red dot in each image; **(B)** the same plot as **(A)** except for cubes with $D_{\text{cube}}=6$ cm. **(C)** trajectories of 10 independent runs for each sphere. The thin blue (thick green) lines are for spheres with $D_{\text{sphere}}=6\;\left(8\right)$ cm. The target position is marked by a black dot. The blue dashed square region indicates an actuation module, which corresponds to the blue shaded region in the first image of **(A)**. **(D)** The distance between the object center X(t) (shape center $X_{c}\left(t\right)$ ) and the target position $X_{\text{ref}}$ is plotted as a function of time in solid (dashed) line. The blue (green) lines are for spheres with $D_{\text{sphere}}=6\;\left(8\right)$ cm; **(E)** the same plot as **(C)** except for cubes. The star markers indicate the end of the trajectories; **(F)** the same plot as **(D)** except for cubes.

The initial and target position of the object are at opposite corners of the robot, and the snapshots of typical transportation processes are shown in Figures 8A,B. The objects can be repeatedly transported to their target positions, showing our closed-loop control method is robust. We collect 10 independent runs for each object, and the trajectories are visualized in Figure 8C and (E) for spheres and cubes, respectively. For the $D_{\text{sphere}}=6$ cm sphere, at the beginning of the transportation, different trajectories are close to each other, as shown by the thin blue lines in Figure 8C. They start to diverge when the object hits the cover of an actuation module, marked by a dashed blue square region. Despite this random disturbance, our closed-loop control method can manipulate the sphere to the target position $X_{\text{ref}}$ , shown by the converging trajectories towards $X_{\text{ref}}$ marked by a black dot. For the $D_{\text{sphere}}=8$ cm sphere, the trajectories diverge less than the smaller sphere. This trend is likely due to the fact that the object-to-actuator size ratio is greater for the larger sphere; hence, the disturbance due to the finite size effect is smaller, and the force model in Equation 6 is more applicable. We also compare the dynamics of the shape center $X_{c}\left(t\right)$ with the object center X(t) , and the relative distance to $X_{\text{ref}}$ as a function of time is shown in Figure 8D. Initially, X(t) remains constant, presumably due to the response time of the actuator and the inertia of the object. Then, the $D_{\text{sphere}}=6$ cm sphere undergoes rapid movement with an average speed of about 8.8 cm/s. Finally, the object is stabilized near the target position, and a constant offset of approximately 0.75 cm persists. This offset is likely due to the fact that steady-state errors cannot be eliminated by the proportional feedback controller we designed in Equation 9. The convergence of $X_{c}\left(t\right)$ is similar to X(t) , and it converges to $X_{\text{ref}}$ around t=2 s, effectively stops the motion of the shape center. For the $D_{\text{sphere}}=8$ cm sphere, the convergence is faster, and the constant offset is also presented.

Similar analysis can be performed for the cubic objects, and the results are shown in Figures 8E,F. The trajectories of cubes are much more scattered than those of the spheres. This can be understood as cubes are more irregular than spheres and experience more random forces during transportation. In addition, the trajectories of the larger cube diverge less than those of the smaller cube. The convergence of the distance to the target position is also less smooth, and sometimes cubes can go away from the target, as shown by the increasing trend in Figure 8F. The $D_{\text{cube}}=6$ cm cube may not converge to a stable final position, as indicated by the nonzero $\left\|X_{c}\left(t\right)-X_{\text{ref}}\right\|$ . This may be due to its small size and shape so that the force disturbance is relatively large.

**FIGURE 9:**
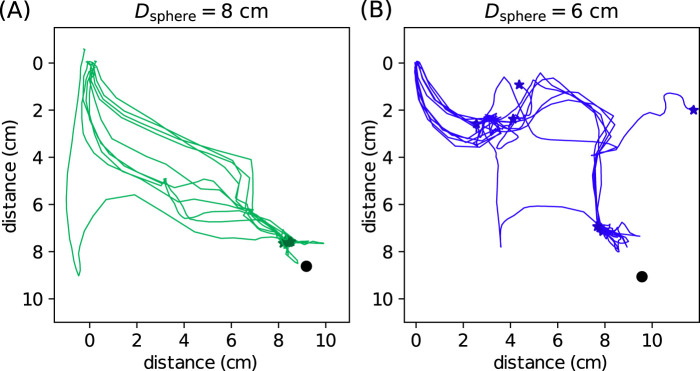
Object transportation experiments without the spherical caps. The target position is marked by a circle, and the end position of each trajectory is marked by a star. **(A)** and **(B)** are trajectories of 10 independent runs for the $D_{\text{sphere}}=8$ cm and $D_{\text{sphere}}=6$ cm spheres, respectively.

To further test the robustness of the design controller, we change the surface property by removing all spherical caps attached to the pins, so the surface becomes flat. This modification increases the randomness during object manipulation, as the objects can sometimes stabilize themselves on the pins or roll away quickly on the flat surface. In addition, the pins are sharper and stiffer, giving greater force disturbance than the soft spherical cap. Without modifying any control parameter, we perform the same object transportation experiments for two spheres and collect 10 independent runs for each object. The resulting trajectories are shown in Figure 9. As expected, the trajectories are more scattered than those in Figure 8. For the $D_{\text{sphere}}=8$ cm sphere, the all 10 tested trajectories can still converge to the target position (the same), while for the $D_{\text{sphere}}=6$ cm sphere, four of ten trajectories fail to get close the target. The closest position is where the $D_{\text{sphere}}=6$ cm sphere falls onto a single pin next to the target position, and that creates a distance gap in Figure 9 because the two positions do not coincide. This failure is likely due to the net effect of small sphere size and the removal of the spherical cap so that the total disturbance is large, and the controller is incapable of transporting the object. We also note that the flat surface makes it difficult to control the two cubes in the current parameter setting. The cubes can sit perfectly on the flat surface, even if the shape is generated; hence, no experimental result is shown. Based on the comparison experiment, it is clear that the spherical caps should be used in practice. This robustness test on the controller suggests that it is still capable of transporting objects under increased disturbance but needs improvements such as additional sensors or advanced feedback methods to estimate and reduce the disturbance when the object stabilizes itself on the surface before reaching the target position.

It is worth noting that our proposed controller does not explicitly require any constraint on the object shape. The ideal object–surface interaction model in Equation 6 considers the object as a point mass, and the robotic surface being smooth. In this regard, a smooth spherical object would be close to the ideal model. In other words, the controller is more applicable to spherical objects. The effects of shape or surface irregularities are shown in Figure 8 or Figure 9, respectively. Irregularities in object shapes and the robotic surface are both considered disturbances and can lead to unstable trajectories, as the interaction force can have greater fluctuations via unstable contacts, rolling, or slipping, etc.

**FIGURE 10:**
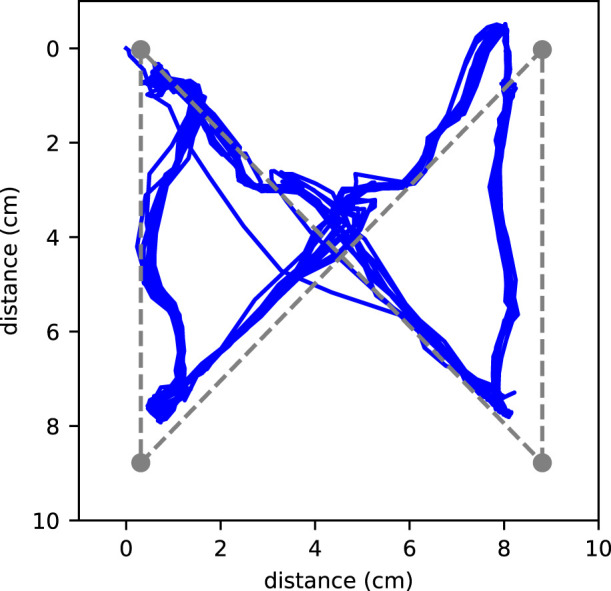
The robot continuously controls the D=8 cm sphere along a bowtie-shaped trajectory.

Finally, to showcase the object manipulation capability of the robot, we have the robot continuously controlling the D=8 cm sphere along a bowtie-shaped trajectory (with the spherical caps installed). The bowtie shape consists of four linear segments, two of which are the diagonals and the other two are the edges of the 4-by-4 pin array, and we let the sphere trace out of the shape 10 times without stopping. The whole trajectory is shown in Figure 10, and except for the beginning of the first time, all trajectories stay close and track out the bowtie shape.

## 4 Discussion

We present a novel control method for robotic surfaces that can substantially reduce the number of independent inputs. The control method has size-independent refresh time and can lead to an effective object manipulation controller when a suitable approximation function is used. We implement the control method in a robotic surface and experimentally confirm the system size-independent refresh time. In addition, the presence of actuator dynamics does not affect this refresh time scaling behavior. Based on the discrete cosine transform and the matching pursuit algorithm, different shapes are efficiently approximated because fewer control messages are required when compared to the standard sequential method. Hence, our control method is more scalable and has the potential to control robotic surfaces with more actuators. Note that the practical upper limit depends on whether the communication technology can reliably broadcast signals to every module in time. Based on our control method, we also provide a modeling method for object manipulation tasks. Using the GRBF as the shape generator, we simplify the complex interaction force with reasonable assumptions based on order-of-magnitude analysis and symmetry considerations and derive a compact feedback controller for object transportation tasks. The validity of the force model and controller is confirmed by the successful transportation of objects of different sizes and shapes.

Because all modules receive the same control message every time step, our control method can update the state of all modules simultaneously. Alternatively, one may design a schedule procedure that updates only relevant modules at a time and hence saves communication bandwidth even if serial communication is used. In this regard, the advantage of our method is that the schedule step is implicitly performed in computing the control message. For example, if only one module is determined to be actuated, a sufficiently small width parameter (such as $\sigma_{t}$ in Equation 2 or Equation 3) will lead to the same control objective, thereby effectively performing the schedule procedure. This is the case in Section 3.2 for the “peak” shape. For cases when the shape of the robot requires changes in a few modules, our method and the schedule-based procedure can perform equally well. However, for complex tasks requiring control over a fraction of the system, our method is more scalable because it uses a system size-independent number of control messages to coordinate the robot. In practice, a hybrid control strategy that can alternate between the scheduled sequential control and a broadcast control method may be suitable to deploy various shapes quickly. Alternatively, one can switch between the DCT and MP algorithms at runtime by setting a threshold value for the fraction of pins that need to have noticeable movements. When the fraction is large, the DCT algorithm is preferable to the MP because it can handle extended patterns. When the fraction is small, MP can be used to actuate a few pins quickly.

As a multi-actuator system, robotic surfaces benefit from a large number of actuators working together to accomplish various tasks, while suffering from the cost and complexity of coordinating many actuators. In essence, our method sends compressed coordination commands to all actuators. A trade-off may exist between the complication due to system size and the complexity of the commands. Although the refresh time scaling is only validated on a small set of actuation modules, and the closed-loop controller is quite simple, we demonstrate its scalable performance. It can be interesting to achieve shape control and object manipulation with distributed control methods, such as designing a sparse state-feedback gain matrix K (Chanfreut et al., 2021; Babazadeh and Nobakhti, 2017), and compare with our method. In our work, each module has its own microcontroller for processing incoming signals, computing function approximations, and performing position servoing. It may also be interesting to design simpler circuits or even mechanical components, such as the fluidic coupler (Jadhav et al., 2023), for the function approximation purposes. Ongoing work focuses on theoretical controllability and closed-loop stability of this control method, as well as more advanced controllers to handle the disturbance when manipulating deformable or irregular geometries.

## Data Availability

The raw data supporting the conclusions of this article will be made available by the authors, without undue reservation.
